# Not Just a Birthmark: A Case Report of a Subtle Port-Wine Stain Heralding Sturge-Weber Syndrome

**DOI:** 10.7759/cureus.96176

**Published:** 2025-11-05

**Authors:** Mohnish Darshan, Sabavath Arun, Harshit Khandelwal, Amber Kumar, Shikha Malik

**Affiliations:** 1 Pediatrics, All India Institute of Medical Sciences, Bhopal, IND

**Keywords:** leptomeningeal angiomatosis, neurocutaneous disorder, pediatric epilepsy, port-wine birthmark, sturge–weber syndrome, subtle presentation

## Abstract

Sturge-Weber syndrome is a rare neurocutaneous disorder due to a somatic GNAQ mutation, which is characterised by facial capillary malformation, leptomeningeal angiomatosis, and possible ocular involvement. Identifying these faint birthmarks is important because even mild skin involvement may indicate significant underlying intracranial vascular malformations and early neurological complications. We report a seven-year-old boy with focal seizures beginning at two years of age, often progressing to generalised tonic-clonic events, and associated with intermittent left-sided headaches. Examination revealed a faint reddish discolouration over the left face, unnoticed previously by the parents. EEG demonstrated left temporoparietal epileptiform discharges, while MRI showed ipsilateral cerebral atrophy, leptomeningeal and pachymeningeal enhancement, choroid plexus enlargement, and cortical calcifications, confirming the diagnosis of Sturge-Weber syndrome. The patient was optimised on antiseizure medication and scheduled for annual ophthalmological surveillance. This case underscores the importance of considering SWS even when facial vascular lesions are minimal, as early recognition facilitates timely seizure management, counselling, and prevention of long-term neurological and ocular complications.

## Introduction

Sturge-Weber syndrome (SWS) is a rare, sporadic neurocutaneous disorder characterised by a facial capillary malformation, leptomeningeal angiomatosis, and ocular abnormalities such as glaucoma [1,2]. It is caused by a somatic activating mutation in the guanine nucleotide-binding protein G(q) subunit (GNAQ) gene, leading to abnormal vascular development in tissues derived from the embryonic forebrain and overlying ectoderm [1]. The incidence of Sturge-Weber syndrome was noted to be approximately 1 in 50,000 live births without significant demographic variations [3].

Neurological manifestations, most commonly seizures, occur in up to 75-80% of patients with brain involvement. Seizure onset is usually within the first year of life, and early, uncontrolled seizures are associated with poorer cognitive outcomes [4]. Other neurological features include hemiparesis, stroke-like episodes, headaches, and developmental delay. Ophthalmic complications, particularly glaucoma, may occur at any age and require long-term surveillance [4].

The risk of SWS is highest when the port-wine birthmark (PWB) involves the forehead or upper eyelid in a large, segmental distribution, corresponding to the embryonic frontonasal prominence [1]. However, even subtle or localised lesions in high-risk zones may be associated with intracranial vascular malformations [1]. In SWS, delayed diagnosis increases the risk of uncontrolled or drug-resistant seizures, progressive cortical damage leading to cognitive decline, stroke-like episodes, and permanent visual impairment.

We report the case of a seven-year-old boy with focal seizures and a subtle, previously unrecognised facial PWB, subsequently diagnosed as SWS. This case underscores the importance of careful skin examination in children with neurological symptoms, even when cutaneous findings are minimal.

## Case presentation

A seven-year-old male presented to our outpatient clinic with a history of seizures beginning at two years of age. The episodes occurred once every 15-20 days, initially involving the right upper and lower limbs, followed by secondary generalisation to all four limbs. Each event lasted one to two minutes and was followed by brief postictal drowsiness. No precipitating factors were identified. For the past two years, the child had also experienced intermittent headaches localised to the left hemicranium, not associated with seizures and often requiring analgesics. There was no history of abnormal behaviour, visual disturbances, neck pain, focal weakness, or sensory loss. Initial treatment at a local hospital included antiseizure medications and multivitamin supplements. The child was referred to our centre in view of persistent seizures and headaches despite medications. He was the firstborn child of non-consanguineous parents, with an unremarkable perinatal history and normal developmental milestones. He attended school regularly, with no evidence of hyperactivity or aggression. Family history was negative for neurological or dermatological disorders.

**Figure 1:**
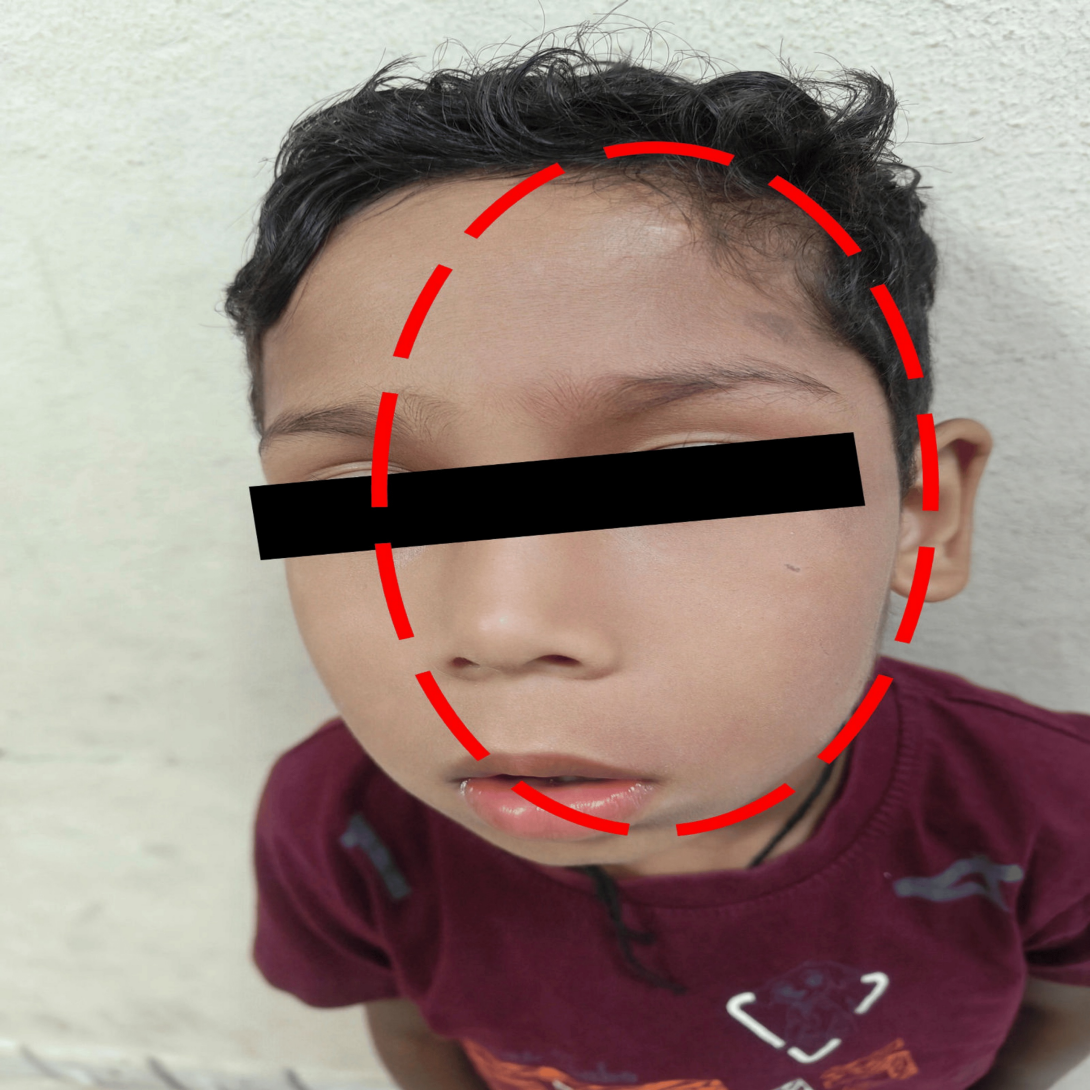
Clinical photograph of the child demonstrating a subtle port-wine stain over the left hemiface along the ophthalmic and maxillary divisions of the trigeminal nerve (outlined in red)

On examination, he was hemodynamically stable: blood pressure 96/60 mmHg (50th-90th centile), pulse rate 96/min, respiratory rate 24/min, and SpO₂ 98% on room air. A subtle reddish discolouration was noted over the left side of the face, consistent with a mild port-wine stain, which had previously gone unnoticed by the parents (Figure 1). The rest of the systemic examination was unremarkable.

Baseline Investigations were within normal limits (Table 1). Electroencephalography (EEG) demonstrated focal epileptiform discharges over the left temporoparietal region. Ophthalmologic evaluation was normal, with no evidence of glaucoma, cataract, or retinal vascular changes.

**Table 1:** Laboratory investigations of the patient in view of seizures with corresponding values and reference ranges

Parameters	Value	Normal range
Haemoglobin	12	12-16 g%
Total leucocyte count	12400	4000-11000cells/mm3
Platelets	254000	150-450 thousand/microL
C-reactive protein	2.4	<5 mg/dl
Urea	19	20-40 mg/dl
Creatinine	0.4	0.6-1.2 mg/dl
Sodium	138	135-145 mmol/L
Potassium	3.9	3-5 mmol/L
Albumin	3.8	3.5-5.5 g/dl
Blood culture	Sterile	-
Urine culture		
Calcium	8.5	8.8-11 mg/dl
Phosphate	4.2	2.5-4.5 mg/dl
Magnesium	2.4	1.9-2.5 mg/dl

**Figure 2:**
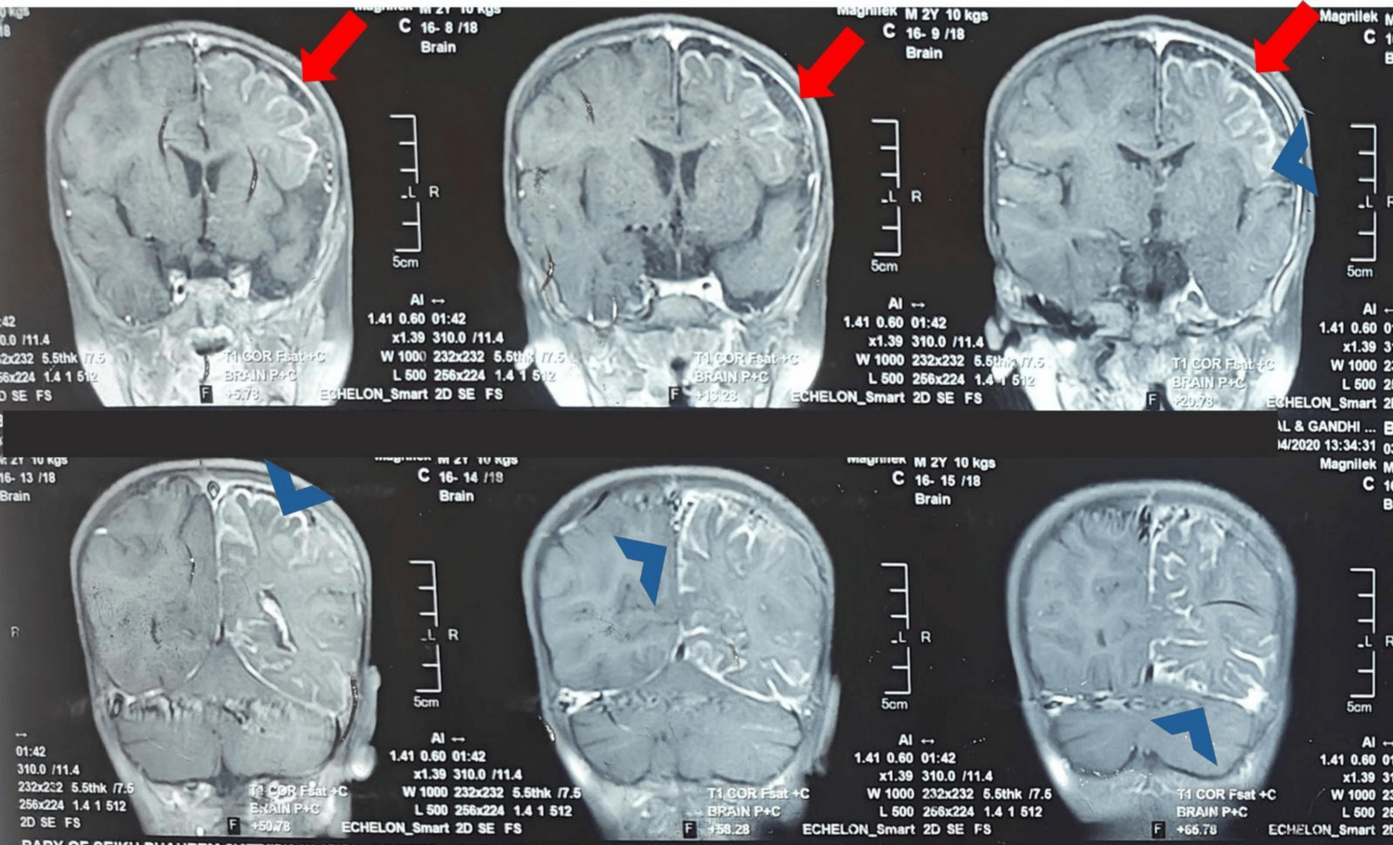
Contrast-enhanced coronal MRI brain images showing leptomeningeal enhancement (blue arrowheads) and underlying cortical atrophy (red arrows) in the left cerebral hemisphere, characteristic of Sturge-Weber syndrome

Given the clinical features, Sturge-Weber syndrome (SWS) was suspected. Magnetic resonance imaging (MRI) of the brain revealed left cerebral atrophy with enlarged sulcal and cisternal spaces, widening of the Sylvian fissure, and diffuse leptomeningeal and pachymeningeal enhancement in the left cerebral hemisphere. There was prominence and enhancement of the ipsilateral choroid plexus and multiple punctate gradient recalled echo (GRE) blooming foci in the left high parietal cortex, likely representing calcifications along dilated cortical veins (Figure 2).

The diagnosis of SWS was confirmed based on the presence of a facial port-wine stain, characteristic imaging findings, and seizure history. The patient’s antiseizure regimen was optimized, and annual ophthalmologic follow-up was advised for early detection and management of possible complications.

## Discussion

Sturge-Weber syndrome (SWS) is an uncommon, sporadic neurocutaneous disorder characterized by the triad of facial capillary malformation, leptomeningeal angiomatosis, and ocular abnormalities. It results from a somatic activating mutation in the GNAQ gene, which disrupts vascular development in the skin, leptomeninges, and choroid [1]. Neurological features, including seizures, hemiparesis, headaches, and developmental delay, are common in patients with cerebral involvement, with seizures reported in up to 75-80% of cases [4]. Early seizure onset and high frequency are associated with worse cognitive outcomes due to cumulative cortical injury from recurrent hypoxia and ischemia [5].

In most instances, SWS is suspected early due to a prominent port-wine birthmark (PWB), particularly when large, segmental, and involving the forehead or upper eyelid. However, as in the present case, subtle or localized PWBs may escape recognition by caregivers and even clinicians, delaying diagnosis until neurological symptoms emerge [6,7]. Although smaller lesions confer a lower risk than extensive hemifacial involvement, the presence of seizures or focal neurological signs in such patients warrants prompt neuroimaging to exclude SWS [3,4,8].

The absence of a facial port-wine stain in Sturge-Weber syndrome (SWS) poses a diagnostic challenge and frequently delays recognition of this neurocutaneous disorder. Such presentations, classified as type III SWS, are rare, accounting for approximately 10% of reported cases [9]. Diagnosis in these patients depends mainly on neuroimaging evidence of leptomeningeal angioma, cortical atrophy, and gyriform calcifications, accompanied by neurological manifestations such as seizures and hemiparesis [10]. In contrast to classical SWS, ocular involvement such as glaucoma is often absent, further obscuring early clinical suspicion [9]. Isolated leptomeningeal angioma without cutaneous features has also been reported in adults, confirming that the characteristic facial nevus is not essential for diagnosis when radiologic and clinical findings are typical [11]. Therefore, early MRI screening in children presenting with unexplained focal seizures or hemiparesis may facilitate recognition of this atypical yet clinically important variant.

Seizures in SWS often have a focal onset corresponding to the side of leptomeningeal involvement and may secondarily generalise [3]. In our patient, right-sided focal seizures matched the left hemispheric pathology seen on MRI. Imaging revealed ipsilateral cerebral atrophy, leptomeningeal enhancement, enlargement of the choroid plexus, and cortical calcifications, which are the findings characteristic of SWS and reflecting chronic venous outflow impairment and hypoperfusion [12].

Although ocular features such as glaucoma are frequently reported, their absence at initial presentation does not exclude future development [4]. Annual ophthalmologic follow-up is therefore recommended to detect and manage these complications early [3]. The importance of early recognition of high-risk PWBs, even when subtle, is emphasised in current consensus guidelines [4]. Early referral to pediatric neurology and ophthalmology facilitates baseline assessment, seizure management, and surveillance for ocular and neurodevelopmental complications [13]. Moreover, while presymptomatic treatment strategies such as antiepileptic therapy combined with low-dose aspirin have been proposed in select high-risk cases, robust prospective data are lacking [4]. Dietary management in Sturge-Weber syndrome aims to maintain adequate nutrition and optimize neurological outcomes. In cases with refractory seizures, a ketogenic or modified Atkins diet may be considered, along with monitoring and supplementation of essential micronutrients such as calcium, vitamin D, and folate [4].

## Conclusions

This case underscores the need for heightened vigilance when evaluating children with even the faintest facial vascular markings. A barely perceptible port-wine birthmark in the forehead or upper-eyelid region can still indicate underlying leptomeningeal involvement, as demonstrated here. Clinicians should perform careful facial inspections during routine examinations, document subtle macules photographically, and maintain a low threshold for neurological and ophthalmological referral whenever seizures, headaches, or developmental concerns arise. Early recognition of such minimal lesions allows timely neuroimaging, anticipatory counseling, and structured follow-up for seizures and glaucoma, helping to prevent the delayed diagnosis and potentially avoidable morbidity seen in this patient.
